# Supplementary material for: Somatic Embryogenesis from the Leaf-Derived Calli of In Vitro Shoot-Regenerated Plantlets of Rosa hybrida ‘Carola’
Source: Plants (Basel). 2024 Dec 19;13(24):3553. doi: 10.3390/plants13243553 (PMC11678673; doi:10.3390/plants13243553)
Supplement: Supplementary file 1 [file plants-13-03553-s001.zip › plants-3316619-supplementary.pdf]

Supplementary Table S1. Effect of different treatments of axillary buds on the germination after 2 weeks of culture in *R. hybrida* 'Carola'.

The type of explants	Germination rate of explants (%)	Average height of axillary buds	Death rate of explants (%)	Contamination rate of explants (%)
Without petiole	100	2.92±0.17	0	6.67
With petiole	100	2.93±0.18	0	26.67

Supplementary Table S2. Effect of different concentrations of 6-BA and NAA on the proliferation after 4 weeks of culture in *R. hybrida* 'Carola'.

6-BA （mg·L-1）	NAA （mg·L-1）	Proliferation coefficient of	Average height of shoot clumps (cm)	Growth state of shoot clumps
0.00	0.00	1.00±0.00 d	0.61±0.09 ef	Young shoos were short and weak
0.50	0.01	1.81±0.18 c	0.84±0.08 de	Young shoots with few leaves and weak
0.50	0.05	1.96±0.18 c	1.04±0.09 cd	Young shoots were dwarfed and slow-growing
0.50	0.10	1.70±0.12 c	0.96±0.11 cd	Young shoots were weak and germinate little calli
1.00	0.01	2.63±0.22 b	0.91±0.09 d	Young shoots in moderate vigor, with light green leaves
1.00	0.05	3.41±0.21 a	1.86±0.11 a	Young shoots shows robust growth with dark green leaves
1.00	0.10	3.44±0.28 a	0.98±0.11 cd	Young shoots were weak, induced calli
1.5	0.01	2.63±0.24 b	1.34±0.06 b	Young shoots were weak, slightly vitrified
1.5	0.05	2.67±0.18 b	1.12±0.05 bc	Young shoots grew well and robust
1.5	0.1	2.78±0.22 b	0.58±0.07 f	Young shoots were weak or even necrotic with abundant calli

Note: Different lowercase letters in the same column indicate significant differences ($p < 0.05$).

Supplementary Table S3. Effect of different concentrations of NAA on the rooting after 3 weeks of culture in *R. hybrida* 'Carola'.

NAA （mg·L-1）	Average number of roots	Average length of roots (cm)	Growth state of roots
0	4.67±0.43 d	0.98±0.07 d	Small number of roots and grew weakly
0.01	5.17±0.47 d	1.44±0.10 c	Roots were strong and grew well
0.05	7.67±0.43 bc	2.52±0.11 a	Roots were stout with well-developed root system
0.1	6.06±0.63 cd	1.72±0.08 b	Roots were strong and grew well
0.2	8.72±0.89 b	0.86±0.07 de	Roots were short and weak
0.3	10.67±0.58 a	0.68±0.07 e	Roots were shorter and weak
0.5	11.85±0.76 a	0.36±0.03 f	Roots were extremely short and weak
1.0	11.11±0.59 a	0.28±0.03 f	Roots were extremely short and weak

Note: Different lowercase letters in the same column indicate significant differences ($p < 0.05$).

Supplementary Table S4. Effect of different hardening-off time on the survival rate after 3 weeks of culture in *R. hybrida* 'Carola'.

Time of hardening-off (d)	Number of plantlets	Number of survival plantlets	The survival percentage (%)
3	18	10	55.56
6	18	17	94.44
9	18	16	83.33
12	18	14	77.78
